# Prediction of arrhythmia after intervention in children with atrial septal defect based on random forest

**DOI:** 10.1186/s12887-021-02744-7

**Published:** 2021-06-16

**Authors:** Hongxiao Sun, Yuhai Liu, Bo Song, Xiaowen Cui, Gang Luo, Silin Pan

**Affiliations:** 1 Qingdao Women and Children’s Hospital, Qingdao University, 266034 Qingdao, China; 2 Institute Oceanology, Chinese Academy of Sciences, 266071 Qingdao, China; 3 Qingdao University of Science and Technology, 266061 Qingdao, China

**Keywords:** Atrial septal defect, Interventional therapy, Random forest, Synthetic Minority Oversampling Technique algorithm

## Abstract

**Background:**

Using random forest to predict arrhythmia after intervention in children with atrial septal defect.

**Methods:**

We constructed a prediction model of complications after interventional closure for children with atrial septal defect. The model was based on random forest, and it solved the need for postoperative arrhythmia risk prediction and assisted clinicians and patients’ families to make preoperative decisions.

**Results:**

Available risk prediction models provided patients with specific risk factor assessments, we used Synthetic Minority Oversampling Technique algorithm and random forest machine learning to propose a prediction model, and got a prediction accuracy of 94.65 % and an Area Under Curve value of 0.8956.

**Conclusions:**

Our study was based on the model constructed by random forest, which can effectively predict the complications of arrhythmia after interventional closure in children with atrial septal defect.

## Background

Atrial septal defect (ASD) is the common congenital heart disease (CHD), accounting for about 10 % of the total CHD, including the following four types, primum, secundum, sinus venosus and unroofed coronary sinus types [1–3]. At present, interventional closure has become the first choice for the treatment of ASD, and the success rate is 97.9-98.7 % [4–6]. The incidence of complications after interventional closure of ASD is 6.3-7.2 %, and arrhythmia is the most common complication [6, 7]. For recent years, people’s interest in artificial intelligence (AI) is increasing. Machine learning (ML), as a subset of AI, is currently widely used in the medical field. ML can build models from dataset and makes predictions, helping experts make accurate predictions and assess disease risks in different environments, so as to develop personalized medical products to achieve precision medicine.

The onset of ASD and the occurrence of postoperative arrhythmias should not be ignored, the length of hospital stay (LOS) will increase, which will affect the healthcare system, especially with the current reduction in beds and increasement of costs [6]. Therefore, accurate prediction of the occurrence of arrhythmias will have a positive impact on health care indicators. Daghistani et al. [8] constructed a model for predicting the length of stay of patients with heart disease, and compared artificial neural networks, support vector machines, Bayesian networks and random forest classification algorithms. Based on the random forest model, the prediction performance was the best, specifically, the sensitivity, the accuracy and Area Under Curve (AUC) were 80 %, 80 % and 0.94, respectively. Przewlockakosmala et al. [9] based on ML, classified and predicted 177 heart failure with preserved ejection fraction patients and 51 asymptomatic control patients on account of rest and post-exercise echocardiography, and identified prognostic phenotypes among patients with heart failure and preserved ejection fraction. Tu et al. [10] built a neural network model based on 15 preoperative factors to predict the long-term hospital stay in ICU after adult cardiac surgery. Bhattacharya et al. [11] constructed an ensemble learning model based on logistic regression and Naïve Bayes to evaluate the risk of ventricular arrhythmia in patients with hypertrophic cardiomyopathy, and obtains a sensitivity, specificity and C-index of 0.73, 0.76 and 0.83, respectively. Alaa et al. [12] used neural networks, support vector machines, random forest, AdaBoost and gradient boosting to build predictive models to assess cardiovascular disease risks. Jalali et al. [13] constructed a deep neural network prediction model to improve the risk prediction of surgical outcome, and the accuracy and AUC value are 0.89 and 0.95 respectively. Luo et al. [14] used weighted support vector machine, weighted random forest and logistic regression to construct a predictive model for congenital heart defects. Based on nine comprehensive variables, weighted support vector machine has the best predictive performance, with the accuracy (ACC), Weighted accuracy (wtACC), AUC and G values of 0.9470, 0.7681, 0.8187 and 0.8088, respectively. Inspired by studies above, this paper proposes a random forest (RF) -based risk prediction model for arrhythmia after interventional closure in children with ASD.

## Methods

### Datasets

The dataset in this article is collected from the clinical dataset of children undergoing interventional closure of ASD at the Heart Center of Qingdao Women and Children’s Hospital from July 2009 to June 2019. All family members of the children signed an informed consent form before the operation. There is a total of 269 children, including 96 males and 173 females. All the patients suffered secundum ASD, and the size of ASD patients with two or more ASD was added as the total defect size. There are 221 patients without arrhythmia, 40 postoperative complications with minor changes, and 8 postoperative complications with major changes. (Table 1) Minor changes includes 32 cases with new incomplete right bundle branch block, 6 cases with I° atrial ventricular block (AV block) and 2 cases with premature atrial contractions. Major changes include 4 cases with frequent premature ventricular contractions, 3 cases with II° AV block and 1 case with accelerated junctional rhythm. Then, due to the imbalance between the data categories, the Synthetic Minority Oversampling Technique (SMOTE) algorithm is used to classify the data. (Table 2) Finally, the data is input into six classifiers to predict postoperative complications, and the prediction performance of the model is evaluated by the leave-one-out method. The comparison shows that the random forest as the classifier has the best prediction effect, with the prediction accuracy rate of 94.65 % and the AUC value of 0.8956.

**Table 1:** Demographic characteristics

	Normal	minor changes	major changes	Total
case number	221	40	8	269
Height (m)	0.94 ± 0.18	0.89 ± 0.20	1.10 ± 0.34	0.94 ± 0.19
Weight (kg)	14.26 ± 6.39	13.28 ± 8.16	26.8 ± 26.5	14.49 ± 8.14
CTR	0.54 ± 0.05	0.55 ± 0.05	0.54 ± 0.03	0.54 ± 0.05
ASD size (mm)	10.21 ± 3.29	10.44 ± 3.00	11.31 ± 5.52	10.28 ± 3.32
LVEF (%)	66.19 ± 2.29	65.98 ± 2.47	64.25 ± 1.98	66.10 ± 2.33
BMI (kg/m^2^)	15.59 ± 1.83	15.88 ± 2.27	17.63 ± 5.04	15.70 ± 2.07
BSA (m^2^)	0.59 ± 0.19	0.55 ± 0.22	0.87 ± 0.60	0.59 ± 0.22
Operating age (year)	3.06 ± 2.02	2.66 ± 2.60	5.50 ± 4.54	3.07 ± 2.25

*CTR* cardiothoracic ratio, *LVEF* left ventricular ejection fraction, *BMI* body mass index, *BSA* Body surface area

**Table 2:** Variables selected for machine learning modeling from the clinical datasets

Input variable	Ranges	Input variable	Ranges
Sex^a^	0–1	Potassium (mmol/L)	3-5.7
Height (m)	0.64–1.30	Sodium (mmol/L)	134–146
Weight (kg)	7–35	CK (U/L)	13.83-276.87
Lung blood^a^	0–1	CK-MB (U/L)	8–43
Precardiac space^a^	0–1	PT (S)	9.8–13.7
CTR	0.4–0.65	INR	0.8–1.19
Right heart enlargement^a^	0–1	APTT (S)	20.1–31.8
ASD size (mm)	5–22	TT (S)	14-119.6
LVEF (%)	60–73	Fibrinogen (mg/dl)	0.97–3.5
Leukocyte (×10^9^/L)	4-13.88	FDP (µg/ml)	2.5-87.94
Erythrocyte (×10^9^/L)	3.5–5.33	D dimer (mg/L)	0-3.02
Hemoglobin (g/L)	86–152	BMI (kg/m^2^)	0.19–14.78
Platelets (×10^9^/L)	100–443	BSA (m^2^)	0.345-1.124
Albumin (g/L)	40-54.98	Operating age (year)	0.50-14.78
ALT (U/L)	5.98–109.6	Creatinine (µmol/l)	4.78-318.69
AST (U/L)	10.21–95.17	Urea (mmol/L)	0.99–7.34

*CTR* cardiothoracic ratio, *LVEF* left ventricular ejection fraction, *BMI* body mass index, *BSA* Body surface area, *ALT* alanine transaminase, *AST *aspartate aminotransferase, *INR* international normalized ratio, *CK* creatine kinase, *CK-MB* creatine kinase-MB, *PT *prothrombin time, *APTT* activated partial thromboplastin time, *TT* thrombin time, *FDP* fibrin degradation products

^a^categorical data. Sex: 0 female; 1 male. Lung blood: normal 0; increase 1; Precardiac space: normal 0; decrease 1. Right heart enlargement: false 0; true 1

### Synthetic minority oversampling technique algorithm

SMOTE algorithm is proposed by Chawla et al. [15], which aims to synthesize some new positive samples to reduce category imbalance. It has been used in drug-target interaction prediction research, protein post-translational sites prediction research and extracellular matrix protein prediction research. The algorithm is briefly introduced as follows:

Given a positive sample *x*, search its nearest neighbor samples *k*, if the oversampling rate is *N*, then select the nearest neighbor sample *N* from the *k* nearest neighbor samples, denoted as *c*_*1*_, *c*_*2*_, … *c*_*N*_, then perform random linear interpolation *c*_*1*_, *c*_*2*_, … *c*_*N*_ between the positive samples *X*, and generate a new positive sample *P*_*j*_ through Eq. (1):

1 $$P_{\mathit j}\mathit=X\mathit\;\mathit+\mathit\;rand\mathit\;\mathit{(0,1)}\mathit\times\mathit{(c_j-X)}\mathit,\mathit\;J\mathit=\mathit\;\mathit1\mathit,\mathit\;\mathit2\mathit,\mathit\;\mathit\dots\mathit\;N$$

Among them, *rand (0,1)* represents the random number in *(0,1)*.

SMOTE is an improved scheme based on the random oversampling algorithm. It is easy to have oversampling problem, which indicates that the information we gain from the model might be too specific, which is not general enough, owing to the fact that random oversampling algorithm is simply to copy samples to increase the minority samples. The basic idea of the Synthetic Minority Oversampling Technique algorithm is to analyze the minority samples and artificially synthesize new samples based on the minority samples and add them to the data set. Steps of the algorithm are as follows:

1. For each sample a in the minority class, use the Euclidean distance as the standard to calculate the distance from all samples in the minority class sample set to obtain its *k* nearest neighbors.
2. Set a sampling ratio to determine the sampling magnification *N* according to the sample imbalance ratio. For each minority sample *a*, randomly select several samples from its *k* nearest neighbors, assuming that the selected nearest neighbor is *b*.
3. For each randomly selected neighbor *b*, construct a new sample with the original sample *a* according to the following formula: *c = a + rand(0,1)∗|a − b|*.

### Random forest

RF, proposed by Breiman [16], is an ensemble learning method based on decision tree classifiers, and has a wide range of applications in bioinformatics. The basic idea is that if there are *N* samples with *M* features in the original training set, RF selects *N* samples from the original training set through Bootstrap resampling, and randomly selects *M* features to train a fully grown tree. Repeat this process to obtain a set of decision tree combinations, summarize their outputs into the integrated model, and vote on the predicted value to generate the final prediction score of RF. Therefore, the number of decision trees and the randomly selected features are critical to build an accurate RF model.

### Support vector machine

Support vector machine was first proposed by Cortes and Vapnik in 1995. It shows many unique advantages in solving small sample, nonlinear and high-dimensional pattern recognition, and can be extended to other machine learning problems such as function fitting.

The support vector machine method is based on the VC dimension theory of statistical learning theory and the principle of structural risk minimization. According to the limited sample information, the complexity of the model (that is, the learning accuracy of a specific training sample) and the learning ability (that is, error-free to find the best compromise between the ability to accurately identify any sample), in order to obtain the best promotion ability (or generalization ability).

### K-Nearest neighbor algorithm

The K-Nearest Neighbor algorithm is one of the efficient and simplest methods for item classification [17]. In KNN, training examples are expressed as points in the feature space in several separate classes. To predict the label of a new item *Ix*, initially, it is projected in the problem feature space. Then, the distances between *Ix* and the *K*-nearest examples are calculated. Then, *Ix* is classified by a majority vote of its neighbors.

### Logistic regression

Logistic model [18] can be applied to regression problems, and also can be used to solve classification problems. In the classification problem, the model can calculate the probability of belonging to each category according to a set of independent variables. Logistic regression model is the most widely used multivariate quantitative analysis method for binary dependent variable.

### AdaBoost

AdaBoost (Adaptive Boosting) is a very popular boosting technique that aims at combining multiple weak classifiers to build one strong classifier. The original AdaBoost paper was authored by Yoav Freund and Robert Schapire.

A single classifier may not be able to accurately predict the class of an object, but when we group multiple weak classifiers with each one progressively learning from the others’ wrongly classified objects, we can build one such strong model. The classifier mentioned here could be any of your basic classifiers, from Decision Trees (often the default) to Logistic Regression, etc.

### Decision tree

Decision tree is one of the predictive modelling approaches used in statistics, data mining and machine learning.

Decision trees are constructed via an algorithmic approach that identifies ways to split a data set based on different conditions. It is one of the most widely used and practical methods for supervised learning. Decision Trees are a non-parametric supervised learning method used for both classification and regression tasks.

Tree models where the target variable can take a discrete set of values are called classification trees. Decision trees where the target variable can take continuous values (typically real numbers) are called regression trees. Classification and Regression Tree (CART) is general term for this.

### Model evaluation

In statistical theory, the leave-one-out method, independent sample test and *K* -fold cross-validation are often used to evaluate the predicting performance of the model. The leave-one-out method directly divides the dataset into two mutually exclusive sets, one of which is used as the training set and the other as the test set. *K* -fold cross-validation randomly divides the dataset into *K* mutually exclusive subsets of similar size. Each time one of them is used as a test sample and *K*-1 are used as a training sample. The cross-validation process is repeated *K* times, the average of *K* times of cross-validation is used as the prediction result of the classifier. In this paper, the leave-one-out method is used to train the model. In order to evaluate the prediction performance of the model, sensitivity, specificity, accuracy and Matthew’s correlation coefficient are used as evaluation index, their definition are as follows:

2 $$Sn=\frac{TP}{TP+FN}$$

3 $$Sp=\frac{TN}{TN+FP}$$

4 $$ACC=\frac{TP+TN}{TP+TN+FP+FN}$$

5 $$MCC=\frac{TP\times TN-FP\times FN}{\sqrt{(TP+FN)(TP+FP)(TN+FP)(TN+FN)}}$$

Among them, *TP* is the number of positive samples predicted to be correct, *FP* is the number of negative samples predicted to be wrong, *TN* is the number of negative samples predicted to be correct, and *FN* is the number of positive samples predicted to be wrong. Sensitivity is the percentage of correct predictions for positive data, and specificity is the percentage of correct predictions for negative data. The value of Matthew’s correlation coefficient (MCC) ranges from − 1 to 1, and the value of Sensitivity, specificity, and ACC ranges from 0 to 1. In addition, Receiver Operating Characteristic is a curve based on the sensitivity and specificity, and AUC is the area under the Receiver Operating Characteristic (ROC) curve. As an indicator of the robustness of the prediction model, the closer the AUC value is to 1, the better the prediction performance of the model is.

## Result

### Comparison of dataset imbalance processing methods

The dataset in this article includes 221 samples without complications and 48 samples with postoperative complications, including 40 cases of minor changes and 8 cases of major changes. The classification prediction performance may get a good overall classification accuracy, but be poor on the minority class samples. And the imbalance of the dataset often causes the prediction results to be biased towards the larger class. However, in many practical problems, the minority samples are more special and important. There is a serious data imbalance between the samples. In order to improve the generalization ability of the classifier and reduce the deviation caused by the imbalance of the dataset, before choosing the appropriate classifier, this article uses the SMOTE algorithm to process the samples to overcome the problem of the imbalance of the dataset. The feature vectors that have been balanced and unbalanced by the SMOTE algorithm are input into the random forest classifier, and the leave-one-out method is used to verify and compare the prediction results, as shown in Table 3.

**Table 3:** Comparison of predict result with No-Synthetic Minority Oversampling Technique and Synthetic Minority Oversampling Technique method on dataset

	Accuracy (%)	Sensitivity (%)	Specificity (%)	MCC	AUC
No-Synthetic Minority Oversampling Technique	81.78	2.50	95.63	-0.0335	0.5665
Synthetic Minority Oversampling Technique	94.65	92.50	94.98	0.7980	0.8956

It can be seen from Table 3 that for the dataset, the prediction model is constructed on the balanced dataset and the unbalanced dataset, and the obtained model evaluation indicators are quite different. In terms of the evaluation index accuracy, the accuracy obtained on the balanced dataset after Synthetic Minority Oversampling Technique processing has a greater advantage than the unbalanced dataset. However, due to the imbalance of the dataset itself, this indicator is used to measure. The pros and cons of the algorithm are not representative. After the dataset is balanced by the Synthetic Minority Oversampling Technique algorithm, the Area Under Curve value increases by 32.91 %. The Synthetic Minority Oversampling Technique algorithm balances the dataset by “synthesizing” the complication samples with minor changes and major changes samples, to increasing proportion in the dataset. Therefore, through the above comparative analysis, after Synthetic Minority Oversampling Technique processing, the prediction performance of the model is significantly improved.

It can be seen from Table 4 that for clinical data random forest algorithm as a predictive classification algorithm, the model has the best predictive performance, with accuracy, Sensitivity, specificity, Matthew’s correlation coefficient and Area Under Curve reaching 94.65 %, 92.50 %, 94.98 %, 0.7980 and 0.8956, respectively. The prediction accuracy, specificity, Matthew’s correlation coefficient and Area Under Curve values are all higher than other classification algorithms. Using the logistic regression classifier, the model has the lowest prediction accuracy, with an accuracy of 78.60 %. The accuracy value of random forest is 16.05 %, 15.72 %, 12.04 %, 9.70 and 5.35 % higher than logistic regression, K-Nearest Neighbor, decision tree, AdaBoost and Support Vector Machine respectively. The Matthew’s correlation coefficient value and specificity value of the random forest classification algorithm are 12.06-37.49 % and 6.56-18.92 % higher than the other five classification algorithms, respectively. From the evaluation indicators Sensitivity, specificity, accuracy and Matthew’s correlation coefficient values, the random forest classifier achieves the best predictive performance.

**Table 4:** The prediction results of different classifiers

Classifier	Accuracy (%)	Sensitivity (%)	Specificity (%)	MCC	AUC
Logistic Regression	78.60	77.50	78.76	0.4231	0.7286
K-Nearest Neighbor	78.93	97.50	76.06	0.5295	0.8219
Decision Tree	82.61	80.00	83.01	0.4927	0.8035
AdaBoost	84.95	87.50	84.56	0.5658	0.7489
Support Vector Machine	89.30	95.00	88.42	0.6774	0.8744
Random Forest	94.65	92.50	94.98	0.7980	0.8956

### Comparison of prediction results of different machine learning algorithms

**Fig. 1:**
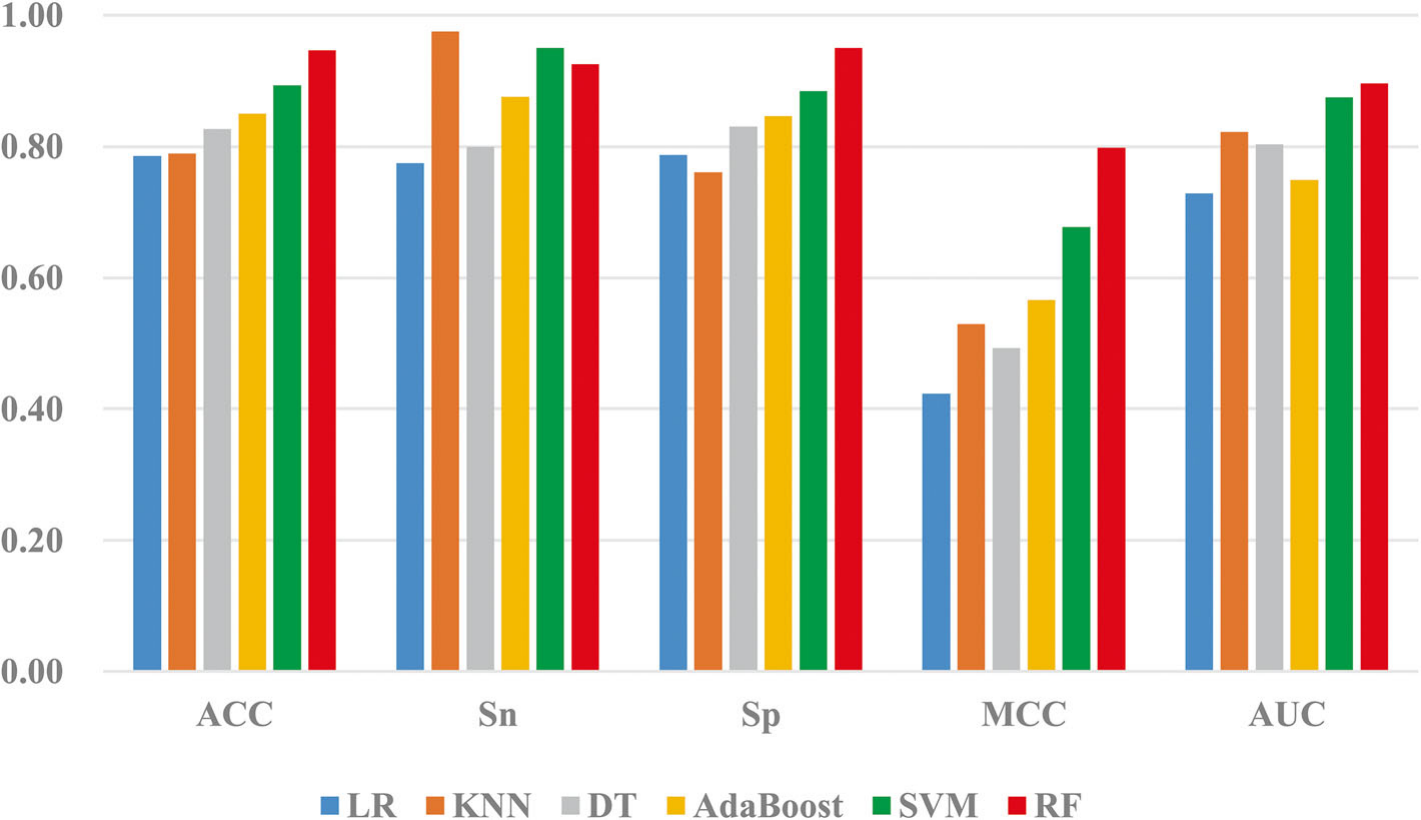
The accuracy (ACC), Sensitivity (Sn), specificity (Sp), Matthew’s correlation coefficient (MCC) and Area Under Curve (AUC) values of different classification algorithms

**Fig. 2:**
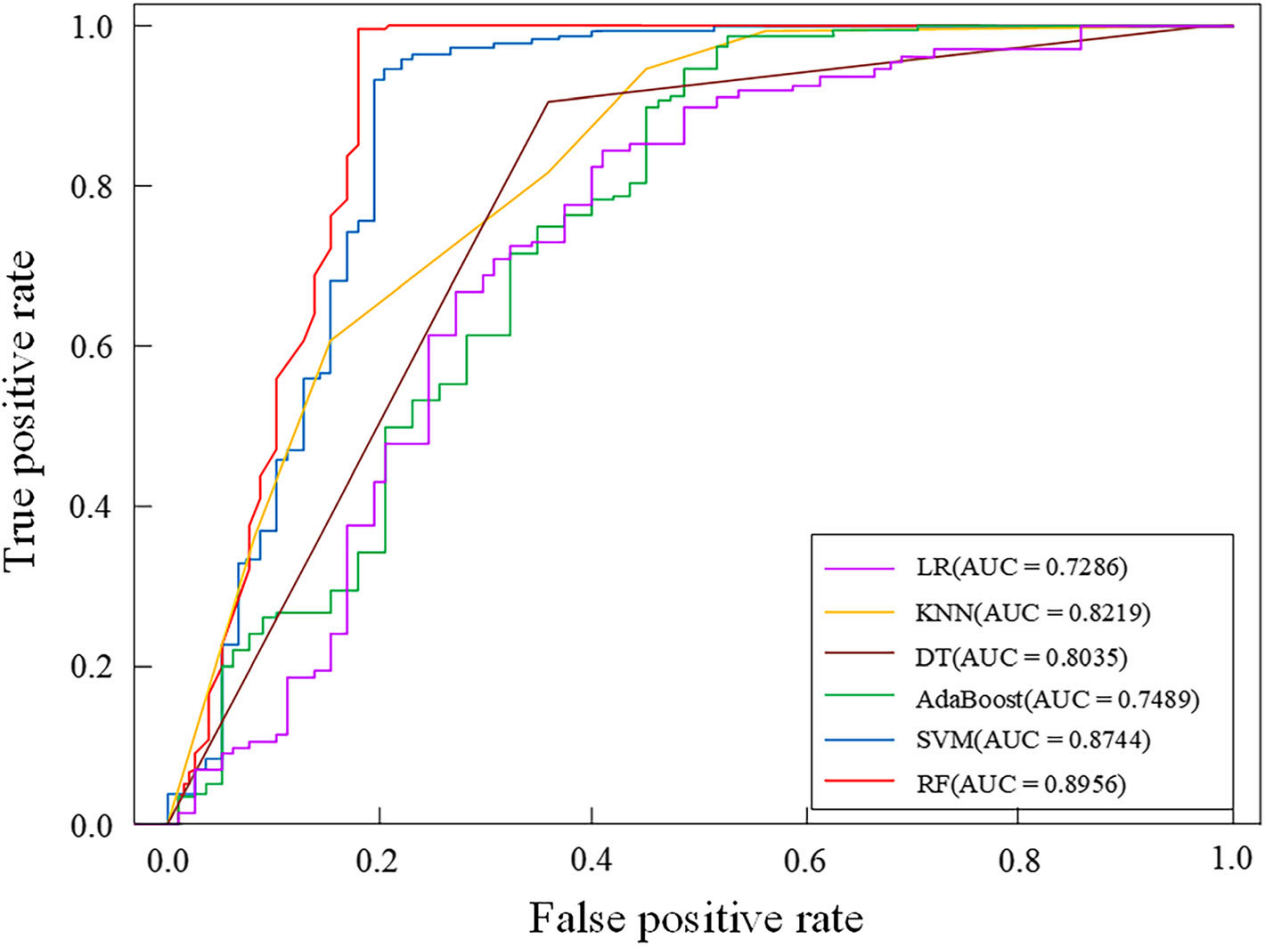
The Receiver Operating Characteristic curves of different classification algorithms

In order to build an efficient prediction model, this paper selects six classification algorithms: logistic regression, *K*-Nearest Neighbor algorithm, decision tree, Support Vector Machine, AdaBoost, and RF to build the prediction model, selects the collected clinical information as the input feature vector, and uses the leave-one-out method to verify the evaluation. The prediction performance of the model and the prediction results of the dataset under different classifiers are shown in Table 4. In order to more intuitively analyze the prediction performance of different classifiers in the training dataset, draw the columns of the ACC value, MCC value and AUC value of the prediction model of complications after interventional closure of children with a ASD under six classifiers, as shown in Fig. 1. In addition, the ROC curve is used to compare the robustness of different prediction models. Figure 2 is the ROC curve obtained by the training set under the six classification algorithms.

It can be seen intuitively from Fig. 1 that the training dataset changes in the ACC, Sensitivity, specificity, MCC and AUC values of the six classifiers logistic regression, K-Nearest Neighbor, decision tree, AdaBoost, Support Vector Machine, and RF. For the ACC, the ACC varies from 78–94 %, the MCC value varies from 0.4 to 0.7, and the AUC value varies from 0.72 to 0.89. Both Support Vector Machine and RF classifiers achieve good AUC values for the training dataset

Choose the Receiver Operating Characteristic curve to compare the prediction performance of different classifiers. If the Receiver Operating Characteristic curve of one classifier is completely enclosed by the curve of another classifier, the prediction performance of the latter is better than the formable. It can be seen from Fig. 2 that for the clinical dataset, the Receiver Operating Characteristic curve of Random forest completely includes the Receiver Operating Characteristic curve corresponding to the classifier’s logistic regression, K-Nearest Neighbor, decision tree, AdaBoost and Support Vector Machine. Its Area Under Curve value is 16.70 %, 7.45 %, 7.37 %, 9.20 %, 14.67 and 2.12 % higher than logistic regression, K-Nearest Neighbor, decision tree, AdaBoost and Support Vector Machine respectively. In summary, Random forest’s Receiver Operating Characteristic curve covers the largest area, indicating that the classification algorithm has the best predictive performance and robustness.

## Discussion

ASD is the third common CHD, accounting for about 10 % of the total incidence of CHD, of which about 70 % are secundum ASD [1–3]. In 1948, Murray [19]closed the ASD under the condition of non-direct vision for the first time, opening the pioneering surgical treatment of ASD. At present, interventional close of ASD has gradually replaced surgery and become the preferred method for the treatment of ASD due to its advantages of less surgical trauma, high success rate, short hospitalization time and low cost. Intraoperative and postoperative arrhythmia is the most common complication of ASD intervention. Due to the stimulation of intracardiac structure by occluders and the push-pull test after the release of occluders, sinus tachycardia, atrial premature beats, atrial tachycardia, AV block and other arrhythmias may occur in some children during the operation. However, most of these children can relieve themselves after surgery, and a few children can last for hours or even weeks. After giving nutritional myocardial drugs or applying glucocorticoid and other treatments, most of them can restore sinus rhythm. Currently, it has been reported in paper that arrhythmias in children with ASD after intervention mainly include sinus bradycardia, sinus tachycardia, supraventricular tachycardia, atrial premature beats, ventricular premature beats, atrioventricular block, atrial fluttering and atrial fibrillation [20–23].

Komar et al. [22] believed that the diameter of the intraoperative occluder was closely related to the occurrence of arrhythmias after occlusion. Large diameter occluders are more likely to cause arrhythmias. ASD is adjacent to the Koch Triangle, and the atrioventricular node is located in the endocardium in front of the Koch Triangle, so it is easy to compress or damage the Koch Triangle by using a large occluder. Jin et al. [24] believed that when the ratio of occluder diameter to ASD size > 0.576, the probability of arrhythmia was significantly increased. The size of ASD determines the type of occluder used intraoperatively. The larger the diameter of the occluder relative to the ASD, the higher the possibility that the edge of the occluder, especially the lower edge, will contact the Koch triangle, and thus the greater the possibility of injury to the Koch triangle. For children with low age, low body weight and large defects, the risk of postoperative arrhythmia is higher than other children due to the shorter length of atrial septum and larger diameter of occlusion device. During the intraoperative release of the occluder and at the early postoperative stage, the occluder may cause compression or friction damage to the surrounding tissues, resulting in corresponding tissue edema and damage, which will affect the conduction function of the electrical pathways of the surrounding heart tissues and cause AV block in the children. However, there are no studies that allow doctors to determine before surgery whether patients will develop postoperative arrhythmias, so as to prevent them early.

AI has become a symbol of the strategic core technology field since its emergence in the 1950 s. In the 1970 s, foreign scholars tried to create a computer algorithm to accurately identify pathological diagnosis [25], opening a new chapter of the rapid development of AI technology in the medical field. AI technologies such as ML can be trained to “learn” different features of data, quantify specific data or correlate with specific diseases [26], and even discover additional predictive information that may not be detectable by the naked eye [27]. Therefore, we constructed a model to prediction of arrhythmia after intervention in children with ASD based on random forest. Available risk prediction models provided patients with specific risk factor assessments, we used SMOKE algorithm and RF ML to propose a prediction model, and got a prediction accuracy of 94.65 % and an AUC value of 0.8956. This prediction model used all 32 variables in Table 2, and played a good role in assessing the risk of postoperative arrhythmias. Although there are many variables used in the model, these variables are all from routine preoperative examinations, and there is no need to add additional examination items, so the workload of clinicians and the economic burden of patients are not increased. Based on this model, we can carry out early prevention for patients with ASD who are at risk of developing postoperative arrhythmias, thus reducing the incidence of arrhythmias after ASD intervention and occlusion. At the same time, we will further screen variables in the following studies in order to obtain fewer and more accurate indicators to predict preoperative arrhythmias.

## Conclusions

This article is based on the model constructed by random forest, which can effectively predict the complications of arrhythmia after interventional closure in children with atrial septal defect. Accurately predicting the risk of postoperative complications and their severity based on preoperative data will help to have more meaningful discussions with family members about the child after surgery. Through the practical application of this model, and after further verification with prospective dataset, we hope to improve clinical decision-making and provide the best predictive information for each family.

## Data Availability

The datasets used and/or analyzed during the current study are available from the corresponding author on reasonable request.

## Abbreviations

ASD: Atrial Septal Defect
CHD: Congenital Heart Disease
AI: Artificial Intelligence
ML: Machine Learning
LOS: length of Hospital Stay
AUC: Area Under Curve
ACC: Accuracy
RF: Random Forest
AV Block: Atrial Ventricular Block
SMOTE: Synthetic Minority Oversampling Technique
CART: Classification and Regression Tree
MCC: Matthew’s Correlation Coefficient
ROC: Receiver Operating Characteristic
